# Rift Valley Fever Virus Incorporates the 78 kDa Glycoprotein into Virions Matured in Mosquito C6/36 Cells

**DOI:** 10.1371/journal.pone.0087385

**Published:** 2014-01-28

**Authors:** Hana M. Weingartl, Shunzhen Zhang, Peter Marszal, Alan McGreevy, Lynn Burton, William C. Wilson

**Affiliations:** 1 National Centre for Foreign Animal Disease, Canadian Food Inspection Agency, Winnipeg, Manitoba, Canada; 2 Department of Medical Microbiology, University of Manitoba, Winnipeg, Manitoba, Canada; 3 Arthropod-Borne Animal Disease Research Unit, United States Department of Agriculture, Manhattan, Kansas, United States of America; George Mason University, United States of America

## Abstract

Rift Valley fever virus (RVFV), genus *Phlebovirus*, family *Bunyaviridae* is a zoonotic arthropod-borne virus able to transition between distant host species, causing potentially severe disease in humans and ruminants. Viral proteins are encoded by three genomic segments, with the medium M segment coding for four proteins: nonstructural NSm protein, two glycoproteins Gn and Gc and large 78 kDa glycoprotein (LGp) of unknown function. Goat anti-RVFV polyclonal antibody and mouse monoclonal antibody, generated against a polypeptide unique to the LGp within the RVFV proteome, detected this protein in gradient purified RVFV ZH501 virions harvested from mosquito C6/36 cells but not in virions harvested from the mammalian Vero E6 cells. The incorporation of LGp into the mosquito cell line - matured virions was confirmed by immune-electron microscopy. The LGp was incorporated into the virions immediately during the first passage in C6/36 cells of Vero E6 derived virus. Our data indicate that LGp is a structural protein in C6/36 mosquito cell generated virions. The protein may aid the transmission from the mosquitoes to the ruminant host, with a possible role in replication of RVFV in the mosquito host. To our knowledge, this is a first report of different protein composition between virions formed in insect C6/36 versus mammalian Vero E6 cells.

## Introduction

Rift Valley fever virus (RVFV), genus *Phlebovirus*, family *Bunyaviridae* is an arbovirus infecting a wide range of mammalian and mosquito species. The virus, endemic to Africa and the Arabian Peninsula, can cause severe disease in humans, and severe often 100% fatal disease in newborn ruminants as well as abortions and mortality in pregnant adult ruminants (e.g. sheep, goats, cattle). RVFV undergoes enzootic and epizootic-epidemic transmission cycles, with *Aedes spp* of mosquitoes being able to transmit the virus vertically, and following heavy rain to initiate epizootic cycles by infecting susceptible livestock (sheep, cattle, goats, camels). Secondary vectors (e.g. *Culex spp*) then contribute to interspecies transmission [1].

**Figure 1:**
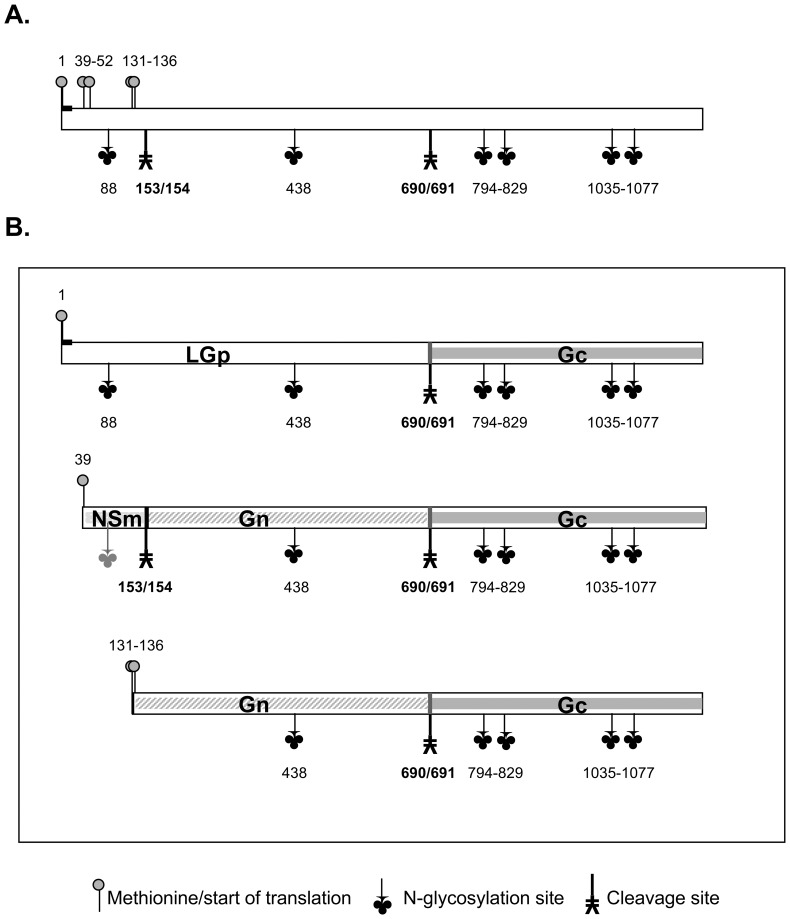
The M segment polyprotein. Schematic summary of the viral proteins expressed from the M segment. **Fig. 1.A.** Translation from the M segment RNA can start at five start codons with methionine in amino acid (aa) positions 1, 39, 52, 131 and 136. All proteins are expressed using the same reading frame. The polyprotein has N-glycosylation sites at aa positions 88, 438, 794, 829, 1035 and 1077, and two cleavage sites between positions 153 and 154, and 690 and 691. The signal peptide (1–16 aa) is represented by a black thicker short line at the very N-terminus. **Fig. 1.B.** Different proteins are generated depending on the start codon used for the protein synthesis. Translation starting with methionine in position “1” yields LGp and Gc glycoproteins due to a cleavage at position 690/691. Both glycoproteins are fully glycosylated. Translation starting with methionine in aa position 39 yields three proteins: nonstructural NSm protein where the N-glycosylation site at aa position 88 may not be utilized, and two glycoproteins Gn and Gc due to cleavage in positions 153/154 and 690/691. No product has been identified for the putative starting methionine in position 52. Translation starting with methionine either at 131 or 136 position yields glycoproteins Gc and Gn, using the 690/691 cleavage site. Gn and Gc glycoproteins are considered to be fully glycosylated. Based on [2], [3], [4].

The virus has a tripartite genome, with one ambisense and two-negative stranded segments. The large segment encodes the RNA-dependent RNA polymerase (L protein), the small (ambisense) S segment encodes the N nucleoprotein and the nonstructural protein NSs. Synthesis and proteolytic processing of proteins encoded by the medium M segment are with five in frame initiation codons and two cleavage sites rather complex [2], [3] (Fig.1.). Based on studies of the RVFV replication cycle in mammalian Vero cells, the M segment can be translated into three M polyproteins depending on the initiation of translation, which upon cleavage yield either two envelope glycoproteins (Gn and Gc) and a nonstructural protein NSm, or the Gn and Gc glycoproteins, or the Gc glycoprotein and a 78 kDa glycoprotein whose function is considered unknown but not essential for virus replication in cell culture [4], [5], [6], [7]. Currently, two glycoproteins (Gn and Gc), nucleoprotein N and polymerase L are considered structural proteins of RVFV [1], [4], [8], [9], [10]. Gerrard and Nichol [4] speculated that since the 78 kDa glycoprotein can form a complex with Gc glycoprotein, similarly to Gn protein, it may be potentially packaged into virions.

RVFV, just as other arthropod-borne viruses, has the ability to efficiently transition from insect to mammalian hosts and to successfully replicate in both. Mechanisms and factors facilitating the transition have yet to be elucidated; however, physical properties of virions may be one of the contributing factors. Differences in the lipid composition of the envelope, the N-glycosylation of the attachment proteins, the configuration of envelope glycoproteins, and the ribonuclear structure between virions matured in mammalian cells versus mosquito cells were detected [11], [12], [13]. To date, differences in protein composition of arboviral virions were not reported.

We considered the possibility that the 78 kDa glycoprotein may be indeed a structural protein, and compared protein composition of virions released from mammalian Vero E6 cells (*Chlorocebus aetiops* origin) to protein composition of virions released from insect C6/36 cells (*Aedes albopictus* origin) with focus on the 78 kDa glycoprotein of wild type RVFV strain ZH501. Because a function of the protein has not been determined yet, and there are differences in reported molecular size, the protein was designated as a “large glycoprotein” (LGp) for the purposes of this work.

## Materials and Methods

### Cells and virus

Vero E6 and C6/36 cells were obtained from American Tissue Culture Collection. Vero E6 cells were maintained in DMEM/10% fetal bovine serum (Wisent) in vent cap flasks (Corning) at 37°C in a 5% CO_2_ incubator. The C6/36 cells were grown in ESF-921 (Expression Systems) medium mixed with EMEM in 1∶1 ratio, supplemented with 10% fetal bovine serum (Wisent)/2.5% HEPES (25 mM final)/1% sodium pyruvate (1 mM final)(Sigma Aldrich)/1% nonessential amino acids (Wisent) at 28°C in phenolic cap or plug seal cap flasks (Corning).

Stock of RVFV strain ZH501, kindly provided by Dr. Heinz Feldmann (National Microbiology Laboratory, Winnipeg), was prepared in Vero E6 cells and plaque titrated as follows: 400 µl/well of tenfold serially diluted samples in DMEM were incubated on confluent monolayers of Vero E6 cells in 12 well plates in triplicates at 37°C in 5% CO_2_ for 1 h. The inoculum was replaced by 1.75% carboxymethyl cellulose (CMC overlay) (Sigma-Aldrich, St. Louis, MO) in DMEM/0.3% BSA (Wisent) supplemented with 25 mM HEPES (Sigma-Aldrich), 100 µg/ml of Streptomycin and 100 IU/ml of Penicillin (Wisent), and incubated for 4 days at 37°C, 5% CO_2_. Formalin (10%) fixed plates were stained with crystal violet (0.5% w/v in 80% methanol in PBS), and virus titer determined in PFU/ml.

### Goat polyclonal anti-RVFV antibodies

The goat RVFV antiserum was developed at NCFAD in goats experimentally infected with RVFV ZH501 [14], and tested for reactivity with individual RVFV proteins using baculovirus expressed recombinant His-tagged proteins: Gc and Gn (developed by S. Zhang), and bacterial recombinant His-tagged N and NSs proteins (kindly provided by J. Jiang, NCFAD), and bacterial recLGp representing the NSm protein plus 38 N terminal amino acids of the M polyprotein (see below).

### Development of antibodies against the 78 kDa large glycoprotein (LGp)

**Figure 2:**
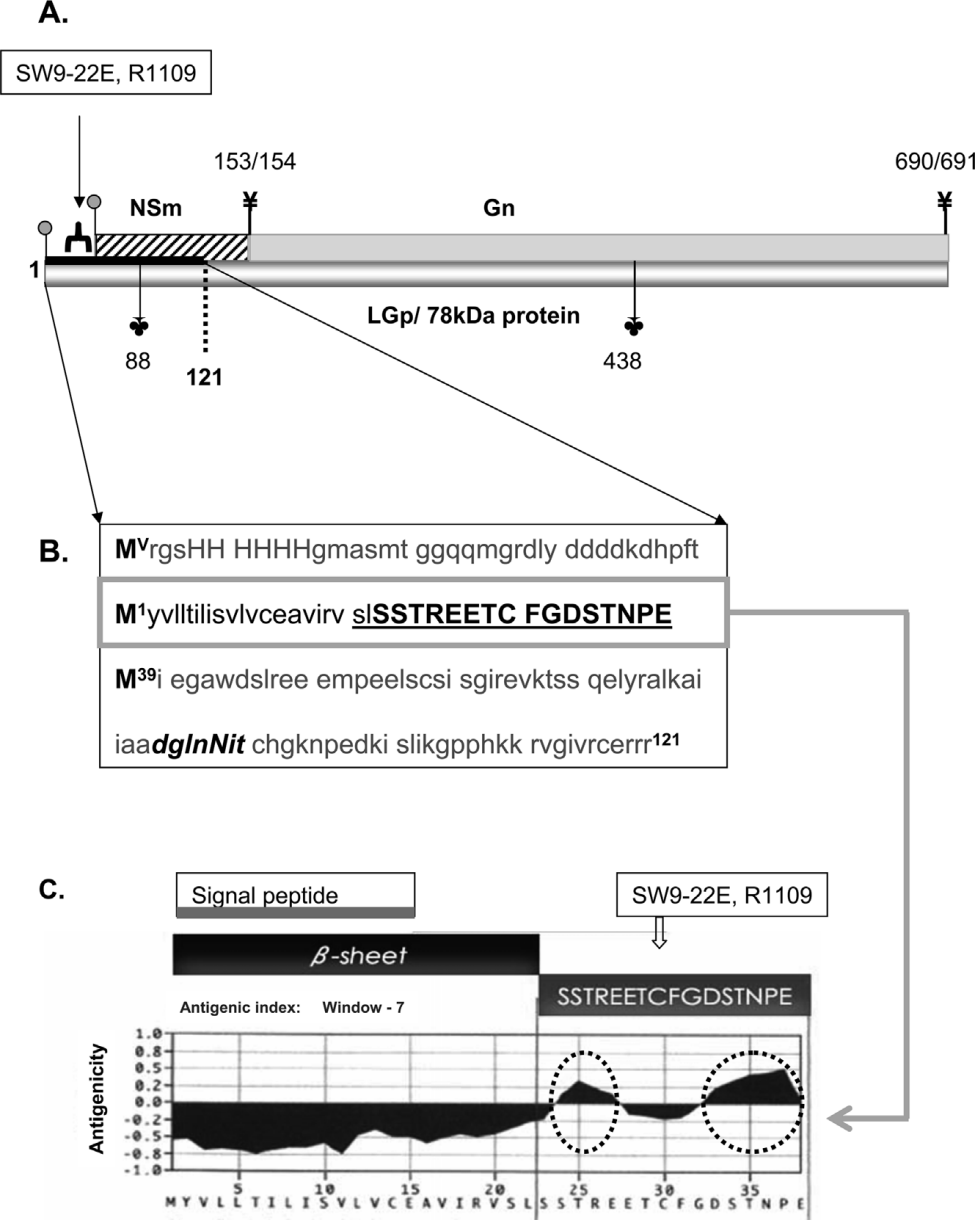
Selection of the peptide for antibody development. **Fig. 2.A.** Schematic representation of the LGp/78 kDa glycoprotein (shaded bottom bar), Gn (gray top bar) and NSm (black striped bar) proteins. Gray full circles on stems represent the methionines in position 1 - start of the LGp/Gc polyprotein, and in position 39 - start of the NSm/Gn/Gc polyprotein. Forks indicate the two cleavage sites 153/154 and 690/691 in the M polyprotein. With translation starting at the methionine in position 39, cleavage at this sites leads to generation of the NSm, the Gn and the Gc proteins. With translation starting at the methionine in position 1, the cleavage occurs only at the 690/691 aa resulting in the LGp and Gc proteins. Clover leaves indicate the glycosylation sites (aa 88 and 438). Based on Gerrard and Nichol [4]. Black solid bar represents the truncated recombinant recLGp (aa 1–121). Small thick fork indicates the peptide region unique to LGp against which the rabbit polyclonal (1109 and 1108) and the mouse monoclonal (SW9-22E) antibodies were raised. **Fig. 2.B.** Amino acid sequence of the recLGp including coding region of the expression vector at the N-terminus. Bold, capital **M** indicates starting methionine (V - in the expression plasmid, 1 - for LGp/Gc, 39 B for NSm/Gn/Gc);string of capital H stands for the His tag; underlined sequence from S to E in capital bold letters indicates sequence of the peptide used for antibody development. Italicized bold sequence ***dglnNit*** represents a potential prokaryotic N- glycosylation signal, and the eukaryotic N- glycosylation signal ***Nit*** (capital N in the 88 aa position). **Fig. 2.C.** Reprint of the EvoQuest predicted antigenicity of the SSTREETCTGDSTNPE peptide (the potential linear epitopes are encircled).

Peptide SSTREETCFGDSTNPE (Fig.2) representing amino acids 23–38 in the N-terminus of the LGp (Nsm1/78/68 kDa) protein was commercially synthesized and used for development of polyclonal rabbit antibodies (R1108, R1109) against this peptide by EvoQuest Team, Invitrogen Corporation (Carlsbad, California). Mouse monoclonal antibody SW9-22E against the same peptide was developed by Open Biosystems, Thermo Fisher Scientific (Huntsville, Alabama).

### Expression of truncated recombinant His-tagged 78 kDa large glycoprotein (recLGp)

In order to confirm reactivity of generated antibodies on immunoblots, cDNA of the LGp (Nt 21 - 384 of the M segment; amino acids 1- 121), representing the unique region of the LGp and the NSm protein was synthesized from the RVFV ZH 501 RNA extracted using TriPure Reagent (Roche). The cDNA was synthesized employing the SuperScript™ III One-Step RT-PCR System with Platinum Taq High Fidelity Polymerase (Invitrogen), forward and reverse primers: CACCATGTATGTTTTATTAACA and TAATCTTCGTCTCTCACACCG, respectively (Invitrogen). Reaction conditions were: one cycle at 50°C for 30 min and 94°C for 2 min, followed by 35 cycles of 94°C for 30 sec, 50°C for 30 sec and 72°C for 40 sec, and final extension at 72°C for 10 min. The fragment was cloned into Champion™ Directional pET200/D-TOPO vector (Invitrogen) with 5 min TOPO clone technique, and chemically competent OneShot TOPO-1 *E.coli* cells were transformed with the resulting plasmid for selection of plasmid with correct nucleotide sequence (Kanamycin selection; PCR screening; sequencing using primers provided in the vector kit). The correct plasmid was transformed into chemically competent BL21 Star™ strain of *E.coli* for IPTG induced expression. Cells were harvested by centrifugation at 18 500 g for 15 min. The pellet was frozen at −80°C prior to purification of the expressed protein with ProBand purification system (Invitrogen) under non-denaturing or denaturing conditions. Briefly, the cell pellets resuspended in binding buffer (8 M urea, pH 7.4) containing Halt™ Protease Inhibitor (Thermo Scientific) were incubated at room temperature for 30 min with rocking, and subsequently sonicated. The expression of the recLGp was verified by an immunoblot of SDS-PAGE separated proteins employing anti-His tag labeled antibodies. Denatured sample in Laemmli buffer (BioRad) was loaded onto NuPAGE precast 4–12% gel in MOPS NuPAGE running buffer. Proteins were separated by electrophoresis at 150 V for 1.5 h, and transferred onto nitrocellulose membrane using iBlot™ dry blotting system (7 min transfer at 20 V) (Invitrogen). The membrane was blocked with 1% alkali-soluble casein (Novagen) for one hour, probed with anti-His antibody (Qiagen Penta-His HRP conjugated antibody, diluted 1∶2000) for one hour at room temperature, and washed three times with 0.1% Tween-buffered saline). Detection was performed using Sigma FastTM 3,3′-diaminobenzidine tablets according to the manufacturer = s instructions. Apparent molecular size of the protein was determined using the SeeBlue^R^Plus 2 Prestained Standard (Invitrogen).

### Protein concentrations

Protein concentration in cell lysates samples was determined by copper-based assay using BCA Protein Detection Kit (Thermo Scientific Pierce). Attempts were made to use the NanoOrange Protein Quantitation Kit (Invitrogen). In the end the amount of protein loaded onto the gels was assessed based on silver stain of protein separation gels. Equal amount of marker was loaded on each gel, and the 28 kDa marker served as a standard to compare amount of RVFV N protein loaded on gels for virion analysis. The ratios of the 28 kDa protein markers to the RVFV N proteins in silver stained protein separation gels were compared by densitometry of corresponding bands using a computer densitometer with the Wright Cell Imaging Facility (WCIF) version of the ImageJ software package http://www.uhnresearch.ca/facilities/wcif/imagej/).

### Deglycosylation

Soluble fraction of the bacterial cell lysate containing the recombinant protein was bound onto the balanced Proband Nickel-Chelating Resin columns (Invitrogen) and eluted with sodium phosphate (200 mM, pH 7.4)/500 mM imidazole/8 M urea buffer (urea was omitted in the native elution buffer). The eluted recLGp was dialyzed (2000 MW cut-off) overnight, and concentrated by using a Centricon column (Millipore; 10,000 MW cut-off). The semipurified recombinant truncated large glycoprotein was treated with PNGase F (QA-Bio) according to the manufacturer's instruction for 3 and/or 24 hrs at 37°C with addition of the Halt™ Protease Inhibitor (Thermo Scientific). The deglycosylation was analyzed by SDS-PAGE followed by immunoblotting.

### Expression of the 78 kDa large glycoprotein protein (LGp) in RVFV infected eukaryotic cells

Cells were scraped off the T75 flasks and together with cell supernatant pelleted down by centrifugation at 2000 g for 10 min. Pellets of uninfected cells or cells infected with RVFV at MOI 0.1 were lysed with I-PER protein extraction buffer containing Halt Protease Inhibitor Cocktail (Thermo Scientific) 24, 48 or 72 hrs post infection, and for the C6/36 cells also at 5 dpi. The proteins were separated by SDS-PAGE as described above, using NUPAGE MES buffer, and transferred onto the nitrocellulose membrane. Blocked membranes were incubated overnight at 4°C with RVFV antiserum diluted 1∶100 which was developed at NCFAD in goats experimentally infected with RVFV ZH501 [14]. Following three washes with Tris buffered saline - 0.1% Tween 20 (TBS-T; Fisher Scientific), membranes were probed with rabbit anti-goat antibody (1∶2000; Jackson) labeled with HRP for 1 h at room temperature, and developed with Fast™ 3,3 = -diaminobenzidine substrate (Sigma).

### Mass spectrometry

Proteins in virion preparations were separated by electrophoresis on the NuPAGE Bis-Tris gels (Invitrogen), and bands in the gel areas corresponding to the 80 kDa molecular size were excised and transferred into Protein Lo-Bind 0.5 ml microcentrifuge tubes (Invitrogen) and gamma-irradiated at 2.5 MRad. Gel slices were cut into 1 mm^3^ cubes and vortexed for 30 min at 700 rpm at room temperature in 1∶1 100 mM ammonium bicarbononate and acetonitrile buffer to destain the gel, dehydrated in 500 µL acetonitrile and air dried, and later rehydrated in 50 µl of 1 M dithiothreitol (Fluka) at 55°C for 30 minutes. Dithiothreitol was replaced with 500 µl acetonitrile for 10 min at room temperature. Acetonitrile was replaced with 50 µl of 50 mM iodoacetamine (Sigma) and incubated in the dark at room temperature for 30 min. Gel plugs were washed twice in 500 µl of 1∶1 100 mM ammonium bicarbonate and acetonitrile buffer (vortexed at 700 rpm for 10 min at room temperature). Gel slices were dehydrated twice in 500 µl acetonitrile for 5 min at room temperature before being dried completely in a SpeedVac. The dried gel slices were rehydrated in 45 µl ice cold 50 mM acetic acid (Sigma) 1.0 µg/µl trypsin (Promega) solution on ice for 2 hrs in the dark. Samples were then incubated at 37°C overnight in the dark. Supernatant was transferred to fresh Protein Lo-Bind 0.5 ml microcentrifuge tubes (Invitrogen). Gel slices were vortexed twice in 100 ul 5% formic acid, 50% acetonitrile solution at 700 rpm for 25 min at room temperature, with supernatant being added to the collection tubes after each vortexing. Supernatant was dried in a SpeedVac and stored at −80°C. Prior to liquid chromatography electrospray ionization tandem mass spectrometry, samples were resuspended in 15 µl of 2% acetonitrile 1% formic acid solution,and analyzed on an LTQ-Orbitrap (Thermo Fisher). Samples were auto-injected via a nano HPLC onto a C18 trapping column and then onto a C18 analytical column (Proxeon) using a 60 min linear gradient 2%–40% ACN in 0.1% formic acid. BSA blanks were run between each sample to reduce protein carryover between samplings. Data were analyzed using Mascot mass spectrometry software (Matrix Science) and viewed using Scaffold3 (Proteome Software).

### Virion preparation for protein analysis

Respective T150 flasks with 95% confluent monolayers of either Vero E6 cells or C6/36 cells were infected with ZH501 RVFV stock generated in Vero E6 cells at an MOI of about 0.1. Virus grown in Vero E6 cells was harvested at 2 dpi, while virus grown in C6/36 cells was harvested at 4 dpi, when titers in the respective cell lines reached around 10^6^ PFU/ml. At this time point, both cell lines also clearly expressed the LGp. Cell culture supernatants only were collected to facilitate the purification of virions. The supernatants were clarified by centrifugation at 10 000 g for 30 min, 4°C. Several approaches were used to gradient purify the virions (employing either iodixanol or sucrose), yielding comparable results. Best preparations were obtained using a following protocol: Clarified cell culture supernatants were concentrated by ultracentrifugation at 150 000 g for 2 hrs through 20% sucrose/TNE (pH 7.4; 0.01 M Tris-HCl; 0.1 M NaCl; 1 mM EDTA) onto 70% sucrose/TNE cushion, and collected in fractions. The fractions were analyzed by real time RT-PCR for RVFV [15] to select the fractions with highest RVFV RNA content for further purification. Collected fractions were diluted with TNE buffer, and the virions were semipurified by ultracentrifugation through a discontinuous gradient of 20, 30, 40, 50, 60 and 70% sucrose in TNE buffer at 140 000 g for 18 hrs, using slow acceleration and slow deceleration (brake turned off at 3000 rpm). The gradient was collected in 1 ml fractions by dripping through the bottom of the tube. RNA concentration in the individual fractions was again determined by real time RT-PCR for RVFV (15), and the fractions with the highest signal were pooled and concentrated by ultracentrifugation at 175,000 g for 18 hrs for protein composition analysis. Sucrose concentration in fractions was monitored using ATAGO Digital Pocket Refractometer (Brix scale).

### Immunobloting

Goat antiserum against RVFV was developed at NCFAD during animal infection experiments [14], and used at 1∶100 dilution for chromogenic detection and 1∶1000 for the chemiluminescent detection. Rabbit polyclonal antibodies (#1109 or #1108) were diluted 1∶100. The mouse monoclonal antibodies SW9-22E specific for the 78 kDa glycoprotein described above were used in 1∶1000 dilution. Affinity purified antibody horseradish peroxidase labeled secondary antibodies were obtained from Kirkegaard & Perry Laboratories: goat anti-rabbit (used in 1∶1000 dilution) and rabbit anti-goat (1∶2000), or from Jackson ImmunoResearch: goat anti-mouse (1∶2000). Protein preparations (cell lysates, virions, recombinant truncated 78 kDa protein) were re-suspended in 1% SDS/PBS, and heat treated at 95°C for 10 min. Protein concentration in the samples was determined using Pierce BCA Protein Assay Kit (Thermo Scientific) read on Spectramax Plus (Molecular Devices). Proteins samples were separated by SDS-PAGE on premade NuPAGE 4–12% Bis-Tris Gel (Invitrogen) in XCell SureLock Mini-Cell Electrophoresis System at 150 V for about 1.5 h employing MOPS or MES running buffer. The proteins were transferred either onto PVDF membranes (Bio-Rad) or nitrocellulose membranes (Invitrogen) using iBlot^R^ dry blotting system (Invitrogen) (7 min transfer at 20 V).

The membranes were blocked with 5% skim milk (BIO-RAD) in 1x TBS-T (0.1% Tween 20) for one hour at room temperature. The membrane was then incubated with primary antibody overnight at 4°C, washed three times with 0.1% TBS-Tween, and incubated with a horseradish-peroxidase (HRP)-conjugated secondary antibody. After three additional washing steps, detection was performed using Fast™ 3,3 = -diaminobenzidine Tablets (Sigma), or using the ECL Detection system (GE Healthcare UK Ltd). Molecular size markers were SeeBlue^(R)^ Plus2 Pre-stained Standard (Invitrogen) or Biotinylated Protein Ladder (Cell Signaling Technology), respectively.

### Sequencing of the RVFV genomic RNA

RNA isolated either from Vero E6 or C6/36 cells derived virus was reverse transcribed and the resulting cDNA amplified using SuperScript III reverse transcriptase in the One-Step RT-PCR System with Platinum *Taq* DNA Polymerase (Invitrogen). The RT-PCR cycle parameters were one cycle at 50°C for 30 min, and 94°C for 2 min, followed by 35 cycles at 94°C for 15 seconds, 56°C for 30 seconds, and 68°C for 2 min, with a final extension at 68°C for 5 min (the S segment). For the M and L segments, the parameters were the same except for the extension times being 4 min, with a final 10 min extension. The entire S and M segments were amplified in one piece, utilizing the following RT-PCR primers: RVFS-Fwd (5′-ACACAAAGCTCCCTAGAGATAC-3′) and RVFS-Rev (5′-ACACAAAGACCCCCTAGTG-3′) for the S segment, and RVFM-Fwd (5′-ACACAAAGACGGTGC-3′) and RVFM-Rev (5′-ACACAAAGACCGGTGC-3′) for the M segment. Amplification of the L segment required two overlapping sections (L1 and L2); these were amplified using: RVFL-L1Fwd (5′-ACACAAAGGCGCCCAATC-3′), RVFL-L1_3482_Rev (5′-GGAAGCATATAGCTGCGG-3′), RVFL-L2_2845_Fwd (5′-GAGACAATAGCCAGGTC-3′), and RVFL-L2Rev (5′-ACACAAAGACCGCCCAATATTG-3′). The RT-PCR products were purified using the Qiaquick PCR purification kit (QIAGEN), and cloned into the pJET1.2/blunt cloning vector using the CloneJET PCR Cloning Kit (Fermentas). Plasmids isolated by QIAprep Spin Miniprep Kit (QIAGEN) were sequenced using the BigDye Terminator v3.1 Cycle Sequencing Kit and the ABI 3130xl Genetic Analyzer (Applied Biosystems). Totals of 7, 12 and 26 sequencing primers were designed for the S, M, and L segment, respectively, to cover the entire segment (available upon request). Sequencing data were analyzed using the DNASTAR LaserGene 9 Sequencing Software.

### Electron microscopy

Concentrated semi-purified virion preparations inactivated with paraformaldehyde were analyzed by electron microscopy. Negative staining: 20 µl of the samples were adsorbed to formvar coated carbon-stabilized copper grids and stained with 2% phosphotungstic acid (w/v), pH 7.2. The specimen grids were examined using Philips CM 120 transmission electron microscope operated at an accelerating voltage of 80 kV, and at nominal instrument magnification of 45,000. Digital images of the virions were acquired by an AMT XR-611-M CCD camera (AMT, Woburn, MA).Immune-electron microscopy: The supernatants containing virions were clarified by centrifugation at 10 000 g for 30 min, 4°C, semipurified by 1 hour ultracentrifugation at 115 500 g for 1 hr through 20% iodixanol/TNE cushion. Virus, resuspended in TNE buffer (pH 7.4; 0.01 M Tris-HCl; 0.1 M NaCl; 1 mM EDTA), was then purified by ultracentrifugation through a discontinuous gradient of 10, 15, 20, 25 and 30% iodixanol in TNE buffer at 210 000 g for 1.5 hr, using slow acceleration and slow deceleration (brake turned off at 3000 rpm), and concentrated at 115 000 g for 2 hrs. Virions were pre-fixed with 1% paraformaldehyde prior to pelleting to preserve their structure. RVFV iodixanol gradient purified virion pellets were fixed in 2% paraformaldehyde/0.25% glutaraldehyde in 0.1 M phosphate buffer (pH 7.2). The pellets were embedded in LR White (London Resin Company LTD.) and polymerized at 50°C for 24 hrs. Nickel formvar-carbon coated grids with 120 nm sections of the RVFV pellet were floated on 0.1% glycine for 10 min, followed by 1% BSA/PBS block for 20 min. The sections were incubated with undiluted SW9-22E hybridoma supernatant, with irrelevant mouse monoclonal antibody or with anti-Gn mouse monoclonal antibody (kindly provided by Dr. Faburay, KSU) at room temperature for 3.5 hrs, blocked again with 1% BSA/PBS, and incubated at room temperature for 75 min with goat-anti-mouse IgG conjugated to 6 nm gold particles (Aurion). The secondary antibody was diluted 1∶10 in 0.1% BSA/0.5% Tween 20/3% NaCl. Grids were washed with PBS prior to fixation in 1% glutaraldehyde for 10 min, then rinsed with MilliQ filtered water, and stained with 2% uranyl acetate for 1 min. The grids were examined using Philips CM 120 transmission electron microscope operated at an accelerating voltage of 80 kV, and at nominal magnification of 45,000. Digital images of the virions were acquired by an AMT XR-611-M CCD camera.

## Results

**Figure 3:**
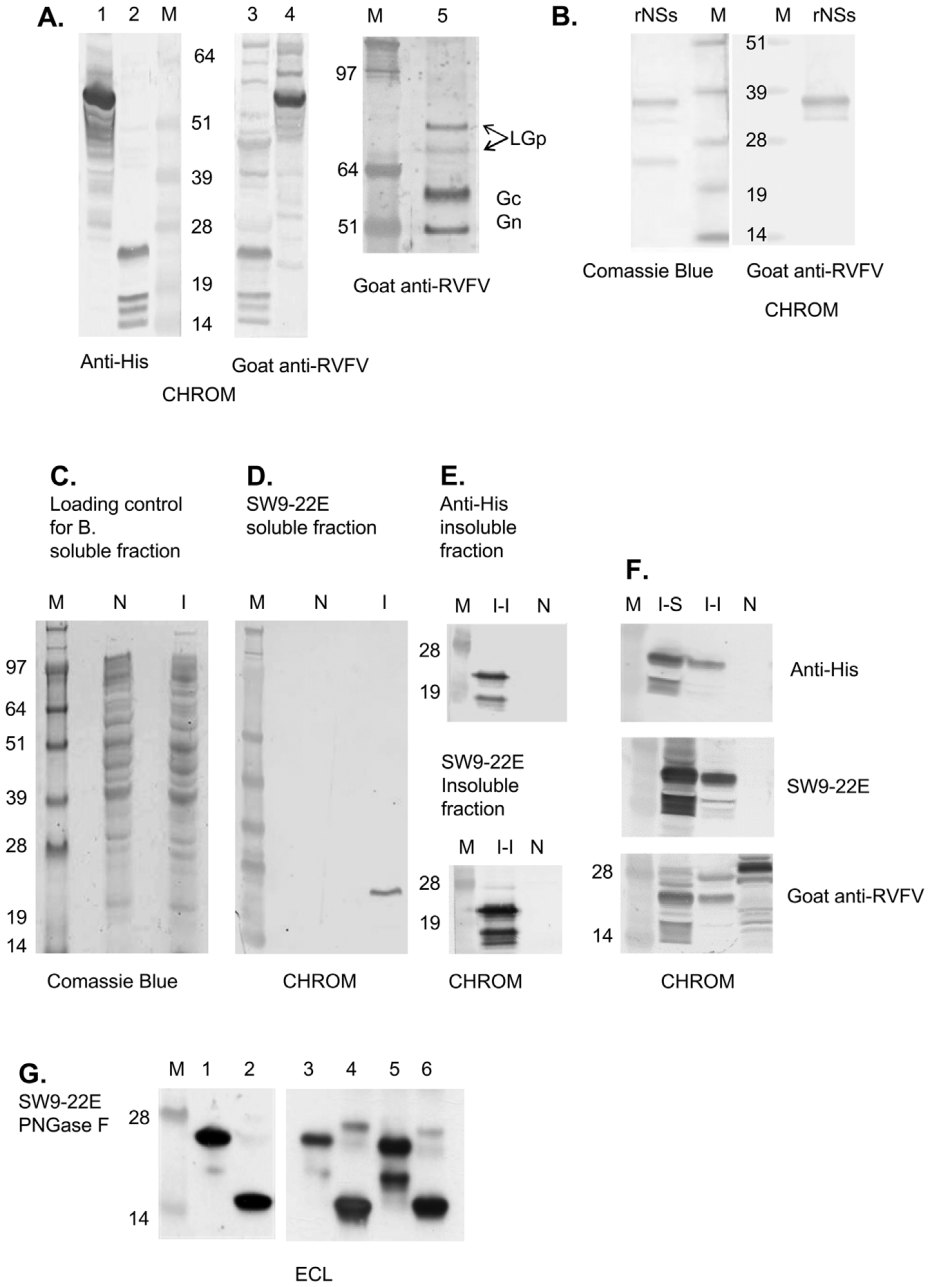
Characterization of antibodies. **Fig. 3.A.** Left panel: Immunoblot of Sf9 cells lysates (soluble fraction) expressing baculovirus recombinant His-tagged Gn protein of RVFV (lanes 1 and 4) and bacterial cell lysate expressing the truncated recombinant LGp containing NSm sequence (lanes 2 and 3) detected with an anti-His antibody (lanes 1 and 2) or goat RVFV antiserum, (lanes 3 and 4). M – marker lane. CHROM  =  Chromogenic detection was used to detect antibody presence. Equal amount of protein (40 µg) was loaded per lane. Right panel, lane 5: Immunoblot of glycoproteins from purified RVFV produced in C6/36 cells using goat anti-RVFV serum. **Fig. 3.B.** Immunoblot of semipurified His tagged recombinant NSs protein of RVFV (400 ng of protein) detected with goat RVFV antiserum; M – marker lane. Left panel – Coomasie blue stained gel, right panel – immunoblot, chromogenic detection. **Fig. 3.C.** Confirmation of the specificity of the SW9-E22 antibody for the rLGp expressed in bacteria. Fig. 3.C is a loading control (Comassie Blue stained protein gel; PAGE was run using MES buffer) for the Fig. 3.D. M lane - protein marker, lane N - 40 µg of proteins from soluble fraction of the bacterial cell lysate, lane I – 40 µg of proteins from soluble fraction of the cell lysate from bacteria with IPTG induced protein synthesis. **Fig. 3.D.** Immunoblot of proteins in soluble fraction detected with SW9-E22 antibody against LGp or goat anti-mouse antibody conjugated with HRP using chromogenic visualization. **Fig. 3.E.** Immunoblot of the induced insoluble fraction from cell lysate of bacteria expressing the recLGp (lane I- I, 40 µg of protein) detected with anti-His antibody conjugated with HRP (top panel) or with SW9-22E antibody (bottom panel) using chromogenic detection. Lane N - transfected, uninduced *E.coli* B21 cell lysate (40 µg of protein). Lane M - protein size markers (SDS PAGE was run using MOPS buffer). **Fig. 3.F.** Immunoblot of the insoluble and soluble fractions from bacterial lysates expressing the truncated recombinant LGp (rLGp) with antibodies against the His tag detected with the mouse monoclonal antibody SW9-22E or with goat RVFV antiserum. M – marker lane, I-I induced insoluble fraction, I-S induced soluble fraction (20 µg of protein per lane), N - noninduced bacterial lysate (40 µg of protein). **Fig. 3.G.** Deglycosylation of the semi-purified recLGp detected with mouse SW9-E22 antibody and goat anti-mouse antibody conjugated with HRP uing chemiluminescent detection (ECL). Lane M - protein size markers (SDS PAGE was run using MES buffer); Lanes 1, 3 and 5 - untreated recLGp (400 ng); Lane 2, 4 and 6 - N-deglycosylated recLGp (400 ng), 24 hrs treatment with PNGase F.

Because of the significant differences in size between LGp (78 kDa), Gn (54 kDa) and NSm (14 kDa) proteins polyclonal antiserum developed in animals infected with RVFV could be used to detect the LGp in virions by immunoblotting of gel separated proteins. Sheep infected with RVFV develop antibodies against Gn, NSm and LGp, as well as against Gc, NSs, and N RVFV proteins [16], although some will develop anti-NSs antibodies only later in the infection and some may develop only very low levels considered as negative [17]. The goat RVFV antiserum used in this study was therefore tested for its ability to recognize RVFV proteins of interest using recombinant proteins (N, Gn, Gc, NSs, NSm). We were able to confirm that the goat antiserum recognized bacterial His-tagged recombinant nonstructural NSm and NSs proteins (Fig. 3.A, left panel; Fig. 3.B). Since the goat RVFV antiserum recognized both, the Gn and the NSm proteins, it was certain that it would recognize the LGp as well (Fig.1, Fig.2). This was confirmed by immunoblotting of the purified RVFV with goat RVFV –antiserum. In the apparent molecular size range of the viral glycoproteins, the antiserum recognized Gc, Gn as well as LGp, indicating presence of two glycosylation forms of this protein in the C6/36 cells (Fig. 3.A, right panel). Interestingly, several protein bands reacted with both the anti-His antibody and with the goat anti-RVFV serum in the truncated recombinant recLGp preparations (NSm protein plus 38 N-terminal amino acids of the M polyprotein) (Fig. 3.A, Fig. 3.E. Fig. 3.F).

Since all proteins generated from the M segment of the RVFV genome are expressed in the same reading frame [2], and consequently Gn and NSm proteins have overlapping sequences with the LGp, only the very N terminus of the LGp was suitable for development of antibodies specific exclusively for this protein within the RVFV proteome (Fig.1., Fig. 2), critical in order to confirm the presence of the LGp in the virions by immune-electron microscopy.

Monospecific rabbit polyclonal antibodies (R1109 and R1108) and a mouse monoclonal antibody (SW9-22E) were custom developed against a short peptide (amino acids 23–38 from the first methionine) at the N-terminus of the LGp (Fig.2). Data generated in the initial stages of the work using the rabbit polyclonal antibodies were confirmed by the mouse monoclonal antibody presented in the manuscript. The ability of the SW9-22E antibody to recognize the LGp was verified by immunoblotting against a truncated recLGp protein (amino acid positions 1 - 121 in the M segment polyprotein) expressed in bacterial system and His-tagged at the N terminus. In agreement with the His-tagged antibodies and the goat RVFV antiserum, the monoclonal antibody SW9-22E also recognized several protein bands on immunoblots (Fig. 3.E, Fig. 3.F, Fig. 3.G) between about 25 kDa and 14 kDa.

The LGp gene carries a potential prokaryotic N-glycosylation signal sequence D/E-X_1_-N -N-X_2_-S/T at the asparagines in position 87/88 [18], [19] (Fig.1.B), and glycosylation at this site would explain the differences in molecular size of the recombinant protein. Deglycosylation using the N-Glycanase (PNGase F) of the semi-purified rLGp indeed resulted in a single product (Fig. 3.G) with size corresponding to the smallest protein band detected on the immunoblots of the recombinant truncated recLGp (as in Fig.3.A).

**Figure 4:**
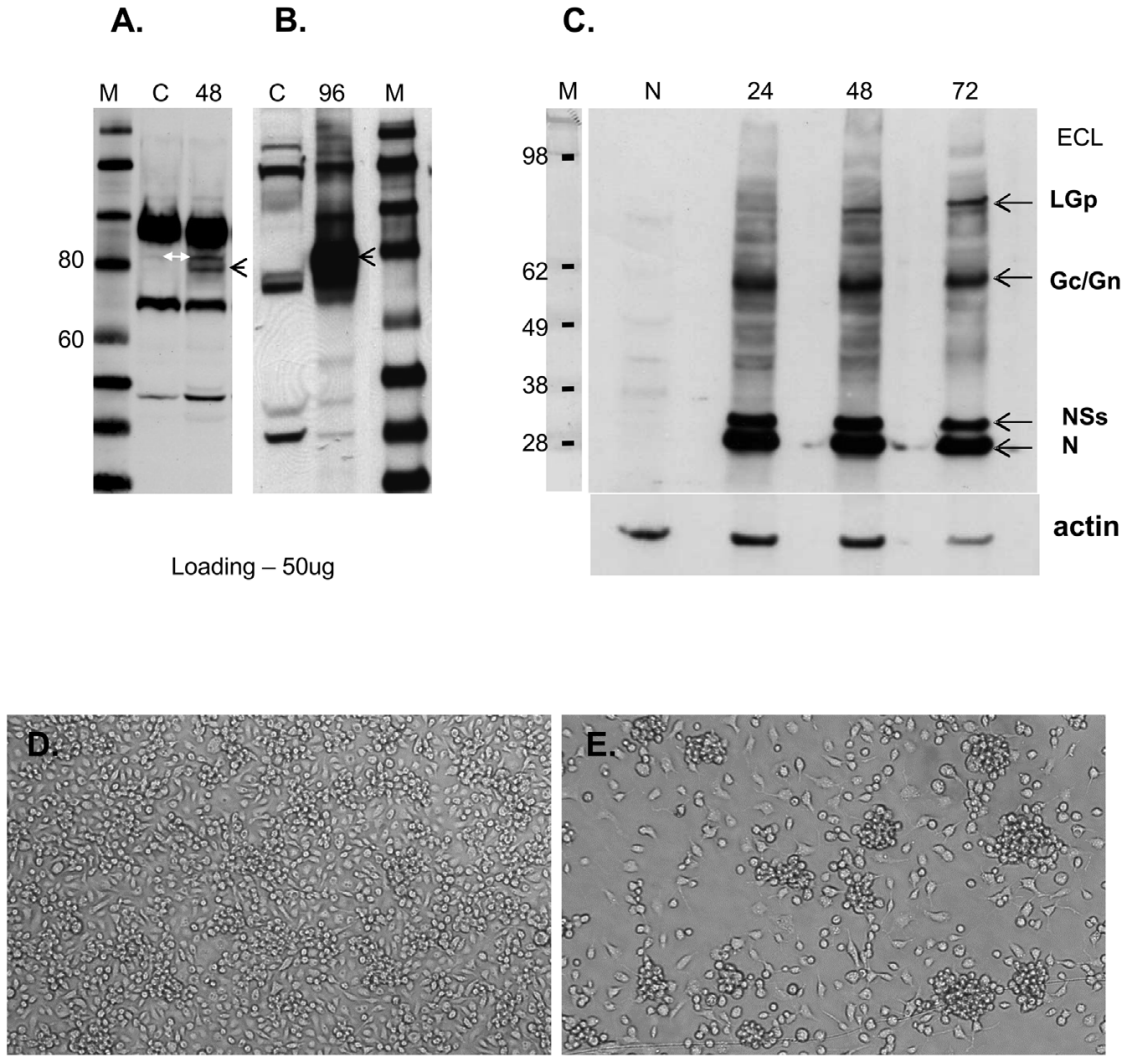
Detection of LGp in cells infected with RVFV. **Fig. 4.A.** Cell lysates of Vero E6 cells detected with the SW9-22E antibody using ECL detection. M- marker lane, C – uninfected cell control, 48 – cells infected with RVFV at 48 hpi. Protein loading 50 µg per lane. **Fig.4** **.B.** Cell lysates of C6/36 cells detected with the SW9-22E antibody using ECL detection. M- marker lane, C – uninfected cell control, 96 – cells infected with RVFV at 96 hpi. Protein loading was 50 µg per lane. Black arrows indicate protein band expected to be the LGp; white arrow indicates a cellular protein upregulated during the RVFV infection. **Fig. 4.C.** Immunoblots of RVFV infected Vero E6 cells with goat RVFV antiserum using ECL detection. This membrane was stripped and re-probed with anti-actin antibody to confirm comparable protein loading in the individual lanes using the anti-actin antibody. M lane indicates the sizes of the protein markers. N lane - uninfected Vero E6 cell lysate negative controls. Lanes 24, 38 and 72 are RVFV infected Vero E6 cell lysates at 24, 48 and 72 hrs post infection (hpi). Protein loading was 50 µg per lane. Weaker detection of actin at 72 hpi is in agreement with expected block of cell protein synthesis during RVFV infection. **Fig. 4.D.** C6/36 cell control, mock infected at 96 h. **Fig.4** **.E.** C6/36 cells infected with RVFV, 96 hpi. Magnification of 40× was used for both figures.

The EvoQuest™ Custom Laboratory Services predicted two potential antigenic sites in the 38 amino acid peptide specific to LGp within the RVFV proteome: SSTREE and DSTNPE (Fig.2.C). It was not possible to exclude that these specific epitopes may be present on proteins within the Vero E6, C6/36 or E.coli proteomes. Indeed, three matches were found for the SSTR epitope within the *Chlorocebus aetiops* proteome using NCBI BLAST (blastp) search. The SW9-22E antibody strongly recognized three proteins and very weakly one additional protein on the immunoblots of uninfected Vero E6 cell lysate (Fig. 4.A.). This protein appeared to be upregulated in the RVFV infected Vero E6 cells (white arrow, Fig.4.A.) at 48 hpi. No match was found for the second epitope. Six annotated proteins carrying the SSTR epitope were identified in the *Aedes albopictus* proteome, roughly corresponding to the number of protein bands recognized on the immunoblot of the unifected C6/36 cells (Fig. 4.B). An additional protein band with molecular size corresponding to the LGp was observed in both cells lines infected with RVFV (black arrowhead, Fig. 4.A and Fig. 4.B). A noticeably higher amount of LGp was detected in C6/36 cells at 96 hpi when the virions were harvested, compared to Vero E6 cells at 48 hrs when the virions were harvested from this cell line. Further confirmation that especially the Vero E6 - RVFV infected cells express detectable amounts of LGp at the time of virion harvest was performed by probing the cell lysates with goat anti-RVFV antiserum (Fig.4.C.). Although Struthers and co-authors [20] reported detection of the radiolabeled 78 kDa glycoprotein (LGp) already at 13 hours post infection (hpi), Besselaar and Blackburn [21] were not able to detect the LGp in infected Vero cell lysate at 24 hpi. In our hands, expression of the LGp was positively confirmed in the Vero E6 cell lysates at 48 hpi (Fig 4.C.). Larger quantities of RVFV proteins, including LGp, appeared to be detected at 72 hpi, however at this point there was already large amount of lysed cells in the supernatant. In contrast the C6/36 cells did not lyse for 7 dpi (maximum tested time), although they were clumping when infected with RVFV (Fig. 4.E).

**Figure 5:**
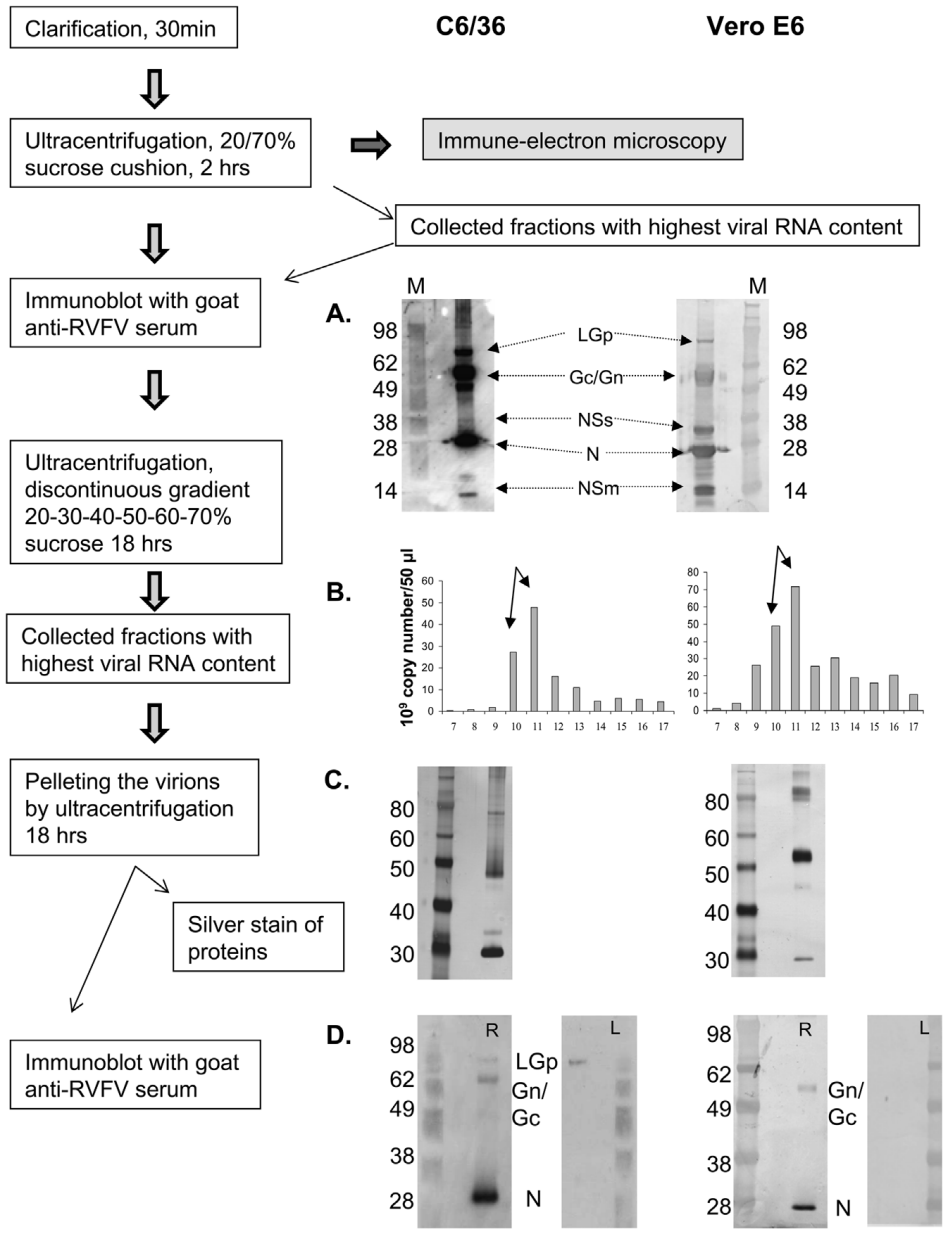
Summary of the virion purification process and an example of virion purification. Purification flow of RVFV virions matured in C6/36 cells (second passage in C6/36 cells) is on the left panels of the figure; purification flow of the Vero E6 matured virions (first passage of C6/36 virus in Vero cells) is on the right panels of the figure. **Fig. 5.A.** Immunoblot using goat antiserum against RVFV of the fractions collected after concentration/semipurification through 20% sucrose onto 70% sucrose cushion. Beside structural proteins Gn/Gc and N, the LGp, as well as nonstructural proteins NSs and NSm were detected in the semipurified virion preparation. The assignment of RVFV proteins to protein bands reacting with the goat RVFV-antiserum was based on expected protein sizes, and known reactivity of the antiserum with respective recombinant proteins. **Fig. 5.B.** RVFV RNA profiles of fractions collected from the discontinuous 20 to 70% gradient (collected from the bottom). Fractions 10 and 11 were then pelleted down and lysed in the loading buffer for the SDS-PAGE. **Fig. 5.C.** Silver stain of the proteins from the gradient purified virion fractions separated by SDS-PAGE. **Fig. 5.D.** Aliquots of the samples from Fig.5.C. analyzed by immunoblotting using goat anti-RVFV serum (left panels designated L) or the SW9-22E antibody (right panels designated R). LGp was detected only in the C6/36 RVFV virions, both virion preparations had detectable levels of structural N and Gn/Gc proteins only.

Virions were harvested at 48 hpi from Vero E6 cells prior to extensive cell lysis, and at 96 hpi from C6/36 cells. These collection time points also corresponded with the time when approximately equivalent infectious virus titers between the two cell culture systems were reached. Virion purification process was for some preparations monitored by immunoblotting of virions semipurified through 20% sucrose cushion before further purification through sucrose gradient. Figure 5. illustrates the purification process where preparations of virions semipurified by ultra-centrifugation through 20% sucrose cushion still contained non-structural proteins in both, C6/36 and Vero E6 prepared RVFV (Fig.5.A.). The preparations for virion protein analysis were further purified. Fractions 10 and 11 containing the highest copy numbers of RVFV RNA were collected from the discontinuous 20 to 70% sucrose gradient (Fig.5.B.), and were after concentration analyzed by silver staining of the separated proteins (Fig.5.C.) and by immunoblotting (Fig.5.D.), using the SW9-22E antibody and the RVFV goat antiserum. Purity of virion preparations following the gradient purification step was considered satisfactory if non-structural NSs protein (and on gels run for shorter period of time also NSm) or cellular proteins were not detected by the goat anti-RVFV antibodies, and silver stain gel of the virions had limited number of protein bands.

**Figure 6:**
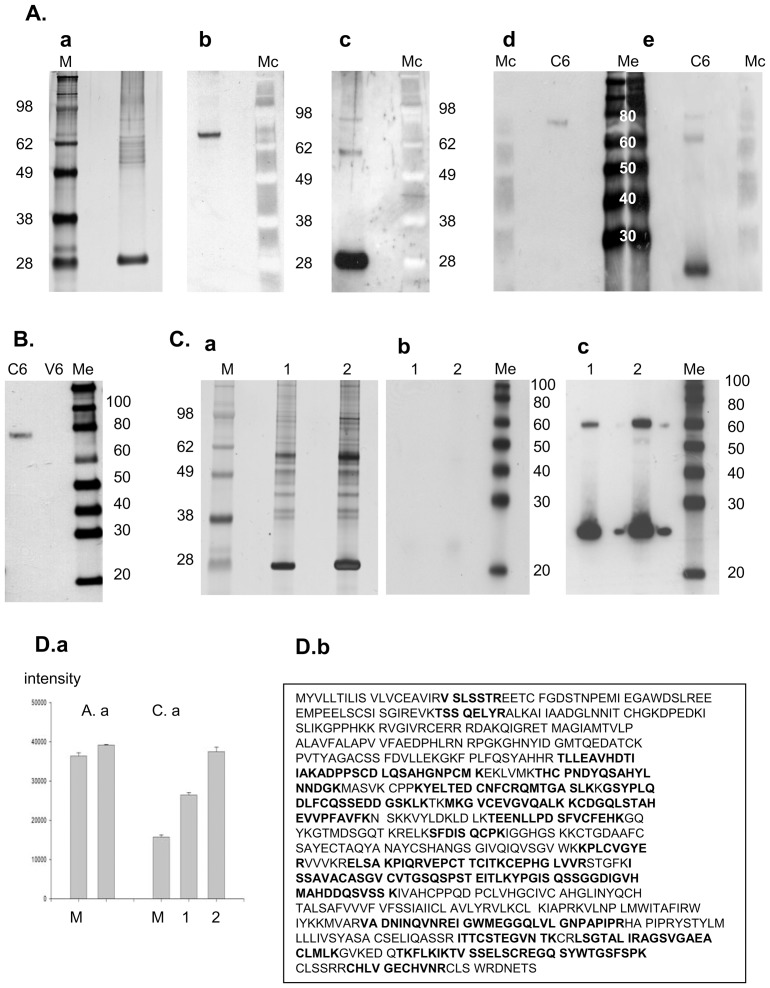
Protein analysis of the gradient purified virion preparations. **Fig. 6.A.** Preparation of RVFV virions grown in C6/36 cells (first passage of Vero E6 virus). **6.A.a** Silver stained denaturing protein separation electrophoresis gel. **6.A.b** Immunoblot of the same sample aliquot as in 6.A.a probed with the anti-LGp antibody SW9-22E. **6.A.c** Immunoblot of the same membrane as in 6.A.b stripped, and re-probed with goat anti-RVFV serum. **Fig.6** **.A.d** Immunoblot of purified RVFV virions matured in C6/36 cells probed with the antibody SW9-22E. **Fig.6** **.A.e** Immunoblot of the same membrane as in 6.A.d stripped, and re-probed with goat anti-RVFV serum to illustrate that in independent samples, the monoclonal antibody SW9-E22 recognized only the smaller form of the LGp while the goat RVFV anti-serum detected only the larger form when used for re-probing the membranes. **Fig. 6.B.** Comparison of the C636 produced virions and the Vero E6 produced RVFV virions. Samples analyzed in Figure 6.A.a and in Figure 6.C.a (lane 1) were analyzed again on the same gel, and probed with anti-LGp antibody SW9-E22. C6 - RVFV virion preparation in C6/36 cells; V6 - RVFV virion preparation in Vero E6 cells. **Fig. 6.C.** Preparation of RVFV virions grown in Vero E6 cells (fourth passage). This preparation yielded double peak on the sucrose gradient, and the peaks were analyzed separately. Sample 1 are fractions collected at around 58% of sucrose and sample 2 at about 53% of sucrose. **6.C.a** Silver stained denaturing protein separation electrophoresis gel. Lane designated as 1 is sample 1, lane 2 is sample 2. **6.C.b** immunoblot of the same sample aliquots as in 6.C.a probed with anti-LGp antibody SW9-22E. **6.C.c** immunoblot of the same membrane as in 6.C.b stripped, and re-probed with goat anti-RVFV serum. Protein separation gels for immunoblotting had both molecular size markers - colorimetric (Mc) and biotin-labeled (Me), and only the ladder closest to samples of interest is presented. **Fig.6** **.D.a** Semi-quantification of the gel loading by measuring intensity of the band for the 28 kDa marker, and the band for the N protein on silver stained gels: A.a. protein densities for Fig.6.A.a - virions produced in C6/36 cells in. C.a protein densities for Fig.6.C.a – virions produced in Vero E6 cells. The intensity comparison indicates that the gels with Vero E6 produced virions were overloaded compare to the gels with C6/36 produced virions, considering that equal amounts of the marker proteins were used in all SDS-PAGE. **Fig.6** **.D.b** Amino acid coverage of the LGp in bold letters as detected by mass spectrophotometry.

In agreement with previously published reports [9], [22], analysis of gradient purified virions from Vero E6 cells did not indicate presence of LGp in the virions. The LGp appeared to be incorporated only into the C6/36 produced virions based on immunoblots with the anti-LGp mouse monoclonal antibody SW9-22E and the goat anti-RVFV serum, and analysis by mass spectrophotometry (Fig. 6). The blots originally probed with the SW9-22E antibody (Fig.6.A.b, 6.A.d. and Fig.6.C.b) were stripped and re-probed with goat anti-RVFV serum confirming the findings. Although the goat RVFV-antiserum was able to recognize two forms of the LGp on the immunoblots (Figure 3.A-right panel), due to re-probing of a membrane originally used for blotting with the monoclonal SW9-22E antibody, only the more abundant, larger form of the LGp was clearly apparent on the blots shown in Figures 5.D/right panel for C6/36 virions and in Figures 6.A.c and 6.A.e. The mouse monoclonal antibody recognized only one, the less abundant smaller, form of the LGp in the C6/36 matured virions (Figure 5.D, left panel for C6/36 virions, and Figures 6.A.b and 6.A.d. Structural proteins of C6/36 derived virions comprised N, Gn/Gc and LGp, and only N and Gc/Gn were confirmed in Vero E6 cell derived virions (Fig.6.A.c and Fig.6.A.e compared to Fig.6.C.c.). Silver staining of protein gels of the virion preparations (Fig.6.A.a and Fig.6.C.a) was beside assessment of purity also used to assess the loading of the gels. Attempts were made to use the NanoOrange Protein Quantitation Kit. Unfortunately, we had to use 1% SDS solution in order to be able to remove the protein virion preparations from the zoonotic AgBSL3 laboratory. This amount exceeded the percentage tolerable by the assay. Intensity of bands on the silver stained gels was used instead. We compared the RVFV N protein quantities based on their ratio to the 28 kDa marker used as a standard. The respective band intensities are recorded in Fig.6.D.a, and indicate higher N protein loading for Vero E6virions in Fig.6.A.a compared to Fig. 6.C.a. The N protein to 28 kDa marker ratio for C6/36 cell matured virions was 1.08, and the ratio for the Vero E6 matured virions in lane 1 was 1.68, while in lane 2 the ratio was higher than 2.5. Although the gel with Vero E6 virions was overloaded, no LGp was observed in virion preparations from this cell line.

In addition, mass spectrophotometry was performed to confirm detection of the LGp in C6/36 virions. Gel slices were excised from protein separation gels of both types of virion preparations in the areas corresponding to the approximately 80 kDa molecular size where positive protein band was detected on the immunoblots with C6/36 matured virions using the mouse monoclonal antibody SW9-E22 or goat anti-RVFV serum. The.analysis by mass spectrophotometry detected 51% coverage of the LGp (353 of 697 amino acids; Fig.6.D.b), including the VSLSSTR sequon unique to LGp in the RVFV, *Chlorocebus aetiops* and *Aedes albopictus* proteomes, and the TSSQELYR sequon shared by LGp and the NSm protein. Total of five samples per C6/36 virion preparations as well as Vero-E6 preparations were analyzed, with only C6/36 virions being positive for presence of the LGp.

Difference in virion composition was observed immediately during the first passage of the virus from Vero E6 cells in the C6/36 cells and vice versa, and detectable virion composition did not qualitatively change from first to second passage of the virus in the same cell line. Figure 5 illustrates virions prepared by second passage of C6/36-RVFV in C6/36 cells and first passage of C6/36-RVFV in Vero E6 cells. Figure 6 illustrates virions prepared by first passage of Vero E6- RVFV in C6/36 cells, and fourth Vero E6-RVFV passage in Vero E6 cells. No differences in the nucleotide sequence of the M segment (and the entire genome) were detected between the virus grown in the Vero E6 cells and the virus grown in the C6/36 cells (data not presented), implying that the changes in virion composition are inherent to the respective cell lines, and do not require any adaptation on the virus part.

**Figure 7:**
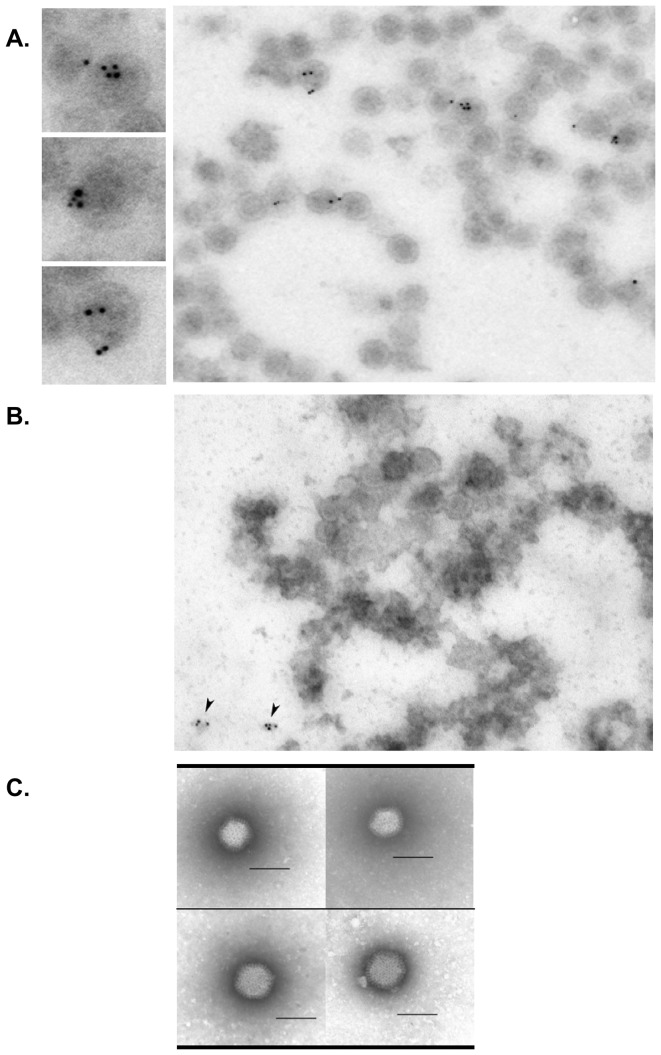
Visualization of the virions by electron miscroscopy. **Fig. 7.A.** Immune electron microscopy of virions produced in C6/36 cells labeled with mouse SW9-22E antibody tagged with 6 nm gold particles with detailed view of selected virions in separate panels. **Fig. 7.B.** Immune electron microscopy of virions produced in Vero E6 cells labeled with mouse SW9-22E antibody. **Fig. 7.C.** Negative staining of virions harvested from Vero E6 cells (upper panels) and negative staining of virions harvested from C6/36 cells (bottom panels). Scale bar represents 100 nm.

Presence of the large glycoprotein LGp on the surface of C6/36 derived virions was further confirmed by immune-electron microscopy in semipurified virion preparations (see Fig. 5), due to difficulties with purification of intact virions, especially the ones produced in Vero E6 cells. Monoclonal antibody SW9-22E detected the LGp only on virions matured in C6/36 cells, as the gold particles were found associated solely with the virion structures (Fig.7.A.). The relatively sparse labeling of the RVFV virions matured in C6/36 cells with the monoclonal antibody SW9-22E may be due to virions having both glycosylation forms of the LGp incorporated into their structure with only the less abundant form recognized by this antibody and labeled with the gold particles. In the Vero E6 matured RVFV virion preparations, the gold particles could be found only occasionally and always associated with debris. (Fig. 7.B.) Attempts were made to label the virions also with anti-Gn monoclonal antibody, unfortunately although the antibody worked well on immunoblots [16] it was not reacting with the virions. The negatively stained C6/36 matured virions (bottom panels) appeared also somewhat larger than the Vero E6 virions (upper panels, Fig. 7.C.). These observations warrant future structural and functional studies, as nothing is known about the actual structure of the virions generated in C6/36 cells, including the ratio of Gn:LGp:Gc incorporated into the virions.

## Discussion

In summary, LGp of wild type ZH501 RVFV was detected in virions maturing in cells of mosquito origin (C6/36), but not in virions matured in cells of mammalian origin (Vero E6), even though both cell lines expressed the LGp. The absence of LGp in virions matured from mammalian-origin Vero E6 cells is in agreement with other reports [1], [4], [8], [9], [10], [22]. Obtaining high purity preparation of virions was difficult as previously reported by others [23], and led to significant losses of material; thus it cannot be excluded that the LGp can be incorporated into mammalian cell derived virions bellow detection limit.

The changes in virion composition were in our experiments inherent to the respective cell lines in which the virions were produced, and did not require adaptation on the virus part, indicating that the differences in virions were cell driven. The considerably higher level of expression of the LGp in the C6/36 cells compared to the Vero E6 cells observed in our experiments at the time of virion harvest may indicate a preference of the insect cells to initiate protein translation from the first methionine, resulting in synthesis of the LGp in quantities allowing efficient incorporation of the protein into virions. This would at the same time result in lower expression of the Gn and NSm proteins. Indeed, Vaughn et al. [24] observed lower expression of the Gn protein in C6/36 cells compared to Vero E6 or the hamster cells BSR-T7/5 cells. Alternatively, as the C6/36 cells do not lyse in course of RVFV replication, and high quantity of the LGp was observed at 96 hpi in the cell lysates, it is possible that the C6/36 cells incorporate the LGp into the virions due to cumulative production of the LGp over the time. A difference in kinetics of virus replication between mosquito C6/36 cells and mammalian Vero E6 and BHK-21 cells with virus replicating slower in the insect cells (this manuscript,[25]) may also account for the higher rate of LGp accumulation, and later harvest of virions. Later harvest was not possible in Vero E6 cells, where the cells started to lyse by 2 dpi when infected with the same MOI as C6/36 cells. Also, the amount of LGp seemed to be significantly lower at the time of virion harvest. At this point, it is not known whether the incorporation of LGp into the C6/36 derived virions is due simply to an abundance of this glycoprotein in insect cells (the signal for assembly is located at the C- terminus, common to both Gn and LGp) or other factors: Differences in lipid composition of the cell membranes between the insect and the mammalian cells may favour incorporation of the LGp into the C6/36 derived virions. Different mode of glycosylation of the LGp in insect cells or other factor(s) may also have an effect on the virion assembly.

RVFV can replicate and form virions in cultured mosquito cells even if the coding sequence for the LGp is deleted from the viral genome. This deletion may have an effect on the virus fitness or virion stability reflected in somewhat lower virus yields compared to mammalian cells [7], [25]. However, LGp may be important for replication in the mosquito host. Crabtree et al., [26] who studied replication of recombinant virus lacking the NSm protein coding sequence in *Aedes aegypti* and *Culex quinquefasciatus* mosquitoes, observed a significantly decreased infection rate and transmission of the recombinant virus compared to the recombinant wild type RVFV. Since deletion of the NSm coding sequence also abolishes expression of the LGp, this observation can be a composite effect of the virus lacking expression of both proteins.

Further investigation of RVFV protein expression and processing during virus replication in insect cells is critical for understanding the natural cycle of the virus, as well as confirming that virions maturing in cultured mosquito cells have the same structure and protein composition as the virions maturing in a mosquito host.

We propose that LGp may be a structural protein in the RVFV virions of mosquito origin, and may represent a strategy to facilitate better transition between different host species, unique to this virus, in addition to a potential role during replication in the insect host. To illustrate other transition strategies of arboviruses, for example, bluetongue virus virion infectivity is enhanced by the saliva of culicoides [27]. Insect derived virus particles of alphaviruses have not only enhanced infectivity but also do not induce IFN- I in primary dendritic cells, due to lipid composition of their envelope [28]. Since dendritic cells are the early target for arboviruses in the skin of the mammalian host, including RVFV [12], efficient first round replication of the infecting virus may thus be secured. A similar effect on plasmacytoid dendritic cells was observed with West Nile virus (flavivirus), where insect derived virions failed to induce IFN-α, while Vero cell-derived virions induced IFN by interaction with endosomal Toll-like receptors [29]. *In vivo*, differences in early replication and immune response were observed in goats and sheep infected with RVFV between inoculum prepared in C6/36 cells versus Vero E6 cells [14]. Previous reports of differences in composition of arboviral virions maturing in mammalian versus insect cells were reported mainly at the level of the envelope lipid composition [10], [11], and the type of glycosylation of the envelope glycoproteins [12]. This is a first report on protein composition of virions of RVFV replicating in mosquito derived cells, as well as a first report on different protein composition of arthropod-borne virus virions between two cell lines, of insect and mammalian-origins.
